# Antihypertensive Drug Use and the Risk of Ovarian Cancer Death among Finnish Ovarian Cancer Patients—A Nationwide Cohort Study

**DOI:** 10.3390/cancers13092087

**Published:** 2021-04-26

**Authors:** Eerik E. E. Santala, Miia Artama, Eero Pukkala, Kala Visvanathan, Synnöve Staff, Teemu J. Murtola

**Affiliations:** 1 Faculty of Medicine and Health Technology, Tampere University, 33520 Tampere, Finland; teemu.murtola@tuni.fi; 2 Finnish Institute for Health and Welfare, 33520 Tampere, Finland; miia.artama@tuni.fi; 3 Faculty of Social Sciences, Tampere University, 33520 Tampere, Finland; eero.pukkala@cancer.fi; 4 Finnish Cancer Registry, Institute for Statistical and Epidemiological Cancer Research, 00130 Helsinki, Finland; 5 Johns Hopkins Bloomberg School of Public Health, Baltimore, MD 21205, USA; kvisvan1@jhu.edu; 6 Sidney Kimmel Comprehensive Cancer Center, Johns Hopkins School of Medicine, Baltimore, MD 21231, USA; 7 Department of Obstetrics and Gynaecology, Tampere University Hospital (TAYS) and TAYS Cancer Centre, 33520 Tampere, Finland; synnove.staff@tuni.fi; 8 Department of Urology, Tampere University Hospital, 33520 Tampere, Finland

**Keywords:** antihypertensive drugs, ovarian cancer survival, risk of cancer death, ACE-inhibitors

## Abstract

**Simple Summary:**

According to the literature, antihypertensive drugs may affect the survival of ovarian cancer patients. We examined the association between different groups of antihypertensive drugs and ovarian cancer survival taking into account dose and duration of use as well as the simultaneous use of other drugs. With a 5-year follow-up, antihypertensive drugs were not associated with survival from ovarian cancer but with 10-year follow-up, ACE-inhibitors and with longer follow-up times also other antihypertensive drugs were associated with improved survival from ovarian cancer. The association between ACE-inhibitor use and ovarian cancer survival should be clarified in the future.

**Abstract:**

Ovarian cancer (OC) has a poor prognosis. Hypertension may be a prognostic factor for OC, but it is unclear whether antihypertensive (anti-HT) drug use of modifies OC prognosis. We performed a population-based analysis assessing the effect of anti-HT drug use on OC mortality. A cohort of 12,122 women identified from the Finnish Cancer Registry with OC in 1995–2013 was combined with information on their anti-HT drug use during the same time period. Use of each anti-HT drug was analysed as a time-dependent variable. Analyses were run for five, ten and full follow-up (19-year) mortality with cardiovascular morbidity risk evaluated in competing risk analysis. No anti-HT drug group was associated with OC survival within five years after OC diagnosis. At ten years, a dose-dependent association was observed between pre-diagnostic ACE-inhibitor use and improved OC survival. With full follow-up, post-diagnostic high-intensity use associated with reduced OC death risk for multiple anti-HT drug groups. In competing risk analysis, only the post-diagnostic use of ACE-inhibitors associated with increased OC survival. Anti-HT drugs were not associated with survival benefits within five years after OC diagnosis. ACE-inhibitors may confer survival benefits in women with OC, but further confirmatory studies are needed.

## 1. Introduction

Ovarian cancer (OC) is the eighth most common cancer and the second most common gynecological cancer after endometrial cancer suffered by Finnish women [1]. It has a poor prognosis with the majority of deaths occurring during the first five years after diagnosis. Subsequently, there is a deceleration in the relative survival rate [2,3]. Unfortunately, the diagnosis of OC is often delayed, and disease prognosis is very poor in comparison to other gynecological cancers [1].

There are multiple histological subtypes of OC but the most common is high-grade serous carcinoma (HGSC) which is often diagnosed at an advanced stage. HGSC is responsible for the majority of all OC deaths and is one of the leading causes of death in women [4,5]. In addition to tumor histology, other prognostic factors are tumor extent, residual disease minimizing cytoreductive surgery, the patient’s performance status as well as genetics such as BRCA-gene positivity which is associated with a slightly improved OC patient survival as compared to those who are BRCA negative [6].

Hypertension is not thought of as an independent risk factor for OC or cancer in general [7,8] but it has been associated with shorter overall survival among women with OC [9]. Antihypertensive (anti-HT) drugs may be beneficial in OC either via the systemic control of hypertension or other cardiovascular diseases or by some unknown beneficial mechanism of action directly against cancer cells. Beta-blockers and especially non-selective beta-blockers have been associated with better OC specific and overall survival [10,11,12,13], though not all investigators have been able to confirm this association [14,15,16]. According to one report, angiotensin-converting enzyme (ACE)-inhibitors, another anti-HT drug group with a different mechanism of action, might improve OC survival [16]. Angiotensin-receptor (ATR)-blockers, but not other anti-HT drugs, have also been associated with a longer recurrence-free time in patients with epithelial OC [17]. In vitro telmisartan, an angiotensin-receptor blocker, was demonstrated to inhibit cell proliferation and induce apoptosis in ovarian cancer cells [18]. The expression level of the AT_1_-receptor (a target of ATR-blockers) in OC cells correlates with poor prognosis and reduced OC survival [19].

In order to estimate effect of any single anti-HT drug group on OC survival, it is essential to take into account the simultaneous use of anti-HT drugs and other drugs some of which may be potential confounders. By adopting this kind of approach, one can evaluate the underlying role of hypertension as it is the common indication of use for all anti-HT drugs; possible confounding by indication (i.e., hypertension) should affect all anti-HT drugs similarly regardless of their antihypertensive mechanism of action. On the other hand, if the risk association should be evident for one particular drug group with a distinct mechanism of action different from the other anti-HT drug groups, this would argue in favor of a molecular effect of this drug group to combat cancer progression. In addition, when evaluating the dose-dependence between drug use and OC survival, the amount of consumed drug should be taken into account although this has not been assessed in previous investigations. Due to the poor prognosis of OC, also the duration of follow-up time might affect risk estimates and should be considered. In previous studies, follow-up times have varied between 6 to 15 years.

Here we have analysed the impact of anti-HT drug use on OC mortality taking into account simultaneous use of multiple drug groups, the cumulative amount of use and also the follow-up time. In order to test whether follow-up time modified the risk associations, we analysed separately five-year and ten-year mortality as well as the risk of OC death with the full follow-up time (maximum 19 years). Our working hypothesis was that the RAA-affecting drugs and beta-blockers might be able to improve the survival of OC patients.

## 2. Materials and Methods

### 2.1. Study Cohort

A cohort of 12,122 ovarian cancer cases was obtained from the Finnish Cancer Registry (FCR) database [20]. The database includes all ovarian cancer diagnoses made in Finland during 1995–2013 via mandatory reports from health care units. The data contain information on the date of OC diagnosis, tumor extent at diagnosis (categorized as localized, locally advanced, distally advanced, advanced to unknown extent, unknown), primary cancer treatment as well as date and cause of death obtained from the national registry of Statistics Finland.

We also obtained the number of 1st degree relatives (children, siblings and parents) from Statistics Finland. These were linked with Cancer Registry to obtain OC and breast cancer (BCa) cases among these individuals. This information was used to estimate the inherited cancer risk, for example due to BRCA 1 and 2 mutations [21]. The number of biological children was assessed because pregnancy and breastfeeding have been associated with a reduced OC risk [22,23]. From the birth date of the first child, we calculated the women’s age at time of first labour, a factor claimed to influence the OC risk [24].

### 2.2. Information on Anti-HT Medication Use

The OC data were linked to the national prescription database maintained by the Finnish Social Insurance Institution (SII) to evaluate anti-HT drug use of the OC patients during the follow-up period 1995–2013. Information on statin and antidiabetic medication use was also obtained to evaluate simultaneous use. SII collects information on all prescribed drugs purchased in an outpatient setting as it provides reimbursements of prescribed drugs for every Finnish citizen. Over-the-counter drugs and drugs consumed in an in-patient setting, for example in hospitals, are not recorded in this database. Information of every purchase includes package size, number of packages bought, dose and the date of purchase.

Information on anti-HT drugs was obtained from the database using unique ATC-codes (Table S1). Anti-HT drugs were divided into six different groups based on their mechanism of action: angiotensin-converting enzyme (ACE) inhibitors (inhibiting ACE), angiotensin-receptor (ATR) blockers (blockade of the AT_1_-receptor), beta-blockers (blocking B_2_- and B_1_-receptor or only B_1_-receptor), calcium-channel blockers (block Ca^2+^-influx in smooth muscle cells), furosemide and other diuretics (both increase diuresis although through different mechanisms). Furosemide was assessed separately because it is not primarily used for the treatment of hypertension.

### 2.3. Statistical Analyses

Anti-HT medication use was determined separately for usage before and after OC diagnosis. The aim was to evaluate the effect of different anti-HT drug use on OC mortality comparing users and non-users. No restrictions by diagnosis of hypertension were made: users of each anti-HT drug group were compared to non-users of anti-HT drugs.

First, a total yearly mg amount for each anti-HT drug for each participant was calculated based on yearly anti-HT drug purchases obtained from the SII-database. Then, the yearly anti-HT drug amount was divided by the drug-specific Defined Daily Dose (DDD) amount to allow a calculation of the total number of doses for each anti-HT drug group [25]. To estimate the use before the diagnosis, DDDs were calculated from 1995 to the year of the OC diagnosis and for the use after the diagnosis, between the year of the OC diagnosis and death/emigration/closing date 31 December 2013, whichever came first.

The intensity of anti-HT drug use was evaluated by dividing the cumulative number of DDDs with the duration of use separately for use before and after the diagnosis.

When estimating the risk of OC death according to the amount of dose, duration and intensity of anti-HT drug use, the participants were divided into three equal size groups based on the cumulative amount of DDDs, duration and intensity of use.

In post-diagnostic analyses, anti-HT medication use was evaluated as a time-dependent variable. User status (non-user/user) was formed for each follow-up year separately. Participants remained as non-users until the year of first drug purchase. All calendar years with any amount of use were recorded as usage years. After anti-HT drug use had been discontinued, participants remained in the user category to avoid bias caused by selective discontinuation of drugs, for example anti-HT drugs are not used in the palliative care of advanced cancer or heart failure [26].

Cox regression analyses was used in the calculation of hazard ratios (HRs) and 95% confidence intervals (CIs) for the association between anti-HT use and the risk of OC death. Follow-up time continued until emigration, death or the closing date of 31 December 2013. All regression analyses were adjusted for the year of the OC diagnosis, age at diagnosis, tumor extent at diagnosis, primary treatments of OC (surgery, cytostatic therapy, radiation or hormonal therapy), number of biological children, age at time of the first labour, number of 1st degree relatives (children, siblings, parents) with OC or breast cancer and use of statins and antidiabetic drugs. All anti-HT drugs were added to the model simultaneously as separate time-dependent variables when evaluating simultaneous use.

Long time associations and the possible role of a protopathic bias were evaluated by lagging the exposure of anti-HT drug use forward for one, three and five years. This means that we estimated the association between the risk of OC death and anti-HT drug use that had occurred for one, three or five years earlier [26]. The data were analysed using the SPSS statistics v. 25 program (IBM, New York, NY, USA).

## 3. Results

### 3.1. Population Characteristics

As expected, due to the dismal prognosis of OC, the median follow-up time in our cohort containing all women was short, 3.3 years and varied between 2.8–4.2 years in the different anti-HT drug groups (Table 1). Anti-HT drug use was common as 7856 ovarian cancer patients had been prescribed at least one anti-HT drug group during the follow-up. Participants using anti-HT medication were older at the time of OC diagnosis and used more often statins and antidiabetic medication in comparison to non-users.

### 3.2. Anti-HT Drug Usage before OC Diagnosis

Regardless of follow-up time, furosemide associated with an increased risk of OC death (Table 2). This elevated risk association was observed with every dose, duration and intensity of furosemide use.

ACE-inhibitors were associated with decreased 10-year OC mortality (HR: 0.92 95% CI: 0.87–0.98) as compared to non-users. Both 5- and 10-year OC mortality declined dose-dependently when the intensity of ACE-inhibitor use increased and thus were lowest for those in group with the highest intensity of ACE-inhibitor use (HR: 0.84 95% CI: 0.77–0.92 at 10 years). No such similar risk decrease or dose-dependence in the risk associations was observed for any other anti-HT drug group. In the full follow-up time (maximum 19 years), high-dose, long duration and high-intensity treatments with beta-blockers and calcium-channel blockers were associated with an increased risk of OC death.

### 3.3. Anti-HT Drug Use after OC Diagnosis

Post-diagnostic ACE-inhibitor use was associated with a reduced OC mortality with full follow-up (HR: 0.81 95% CI: 0.71–0.93) (Table 3). The use of ATR-blockers also associated with decreased OC death risk but only for 10-year mortality. In contrast, consumption of other anti-HT drugs was not associated with a decreased OC death risk.

A dose-dependence between ACE-inhibitor use and OC death was observed: the risk of OC death was lowest in those individuals with the highest intensity use of ACE-inhibitors. However, a dose-dependence was observed also among all other anti-HT drugs regardless of follow-up time as the risk of OC death decreased along with increasing intensity of anti-HT use. Nonetheless, risk estimates remained elevated as compared to non-users with the exception of high-intensity use of ACE-inhibitors, ATR-blockers, beta-blockers and calcium-channel blockers with full follow-up (Table 3). Furosemide and diuretics were associated with an increased risk of OC death with all follow-up times but especially with low intensity use.

In the lag time analyses, a decreased risk of OC death was seen in the ACE-inhibitor group even 5 years after use with full follow-up (Table 4). No such similar direction lag in risk associations was observed for any other anti-HT drug group; the risk associations vanished with even a 1-year time lag. The risk increase among furosemide was especially evident with the 1-year time-lag but then was attenuated to a non-significant level (Table 4).

### 3.4. Sensitivity Analyses

We also ran competing risk analysis where deaths due to cardiovascular diseases were analysed as the competing cause of death (Table S2). In this analysis, post-diagnostic use of ACE-inhibitors continued to be associated with a lowered risk of OC death (HR: 0.73 95% CI: 0.58–0.91) similar to the main analysis with the full follow-up. However, for all other anti-HT drug groups, the risk associations were no longer statistically significant and none of the anti-HT drug groups, with the exceptions of diuretics and furosemide, were associated with an increased risk of OC death.

## 4. Discussion

In our cohort study, follow-up time clearly modified the risk association between anti-HT drug use and ovarian cancer death. None of the drug groups were associated with 5-year mortality, with the possible exception of diuretics. A decreasing risk trend among users of ACE-inhibitors and also among users of other anti-HT drugs in post-diagnostic use was only observed when the follow-up time was ten years or longer. This indicates that the control of hypertension may have prognostic benefits in OC, as the use anti-HT drugs was associated with survival regardless of the drug’s mechanism of action. However, in sensitivity analysis, where cardiovascular morbidity was analysed as a competing cause of death, only ACE-inhibitors were associated with a significantly improved OC survival. Therefore, our study provides some support for the proposal that ACE-inhibitors confer oncological benefits in women with OC.

OC has an overall poor prognosis; in Finland, the five-year relative survival was 44% in the time period 2016–2018 [1]. Among women over 75 years old, the five-year relative survival was less than 25% [1]. This may explain why follow-up time exerts such an impact on the results. Most deaths in OC occur within a few years after the diagnosis. Those women still alive five years after diagnosis likely had either a localized or a less aggressive subtype of disease at diagnosis, when curative-intent treatment is still possible. This may reflect their more active contact with health care professionals, which in turn may include also better management of hypertension and other chronic conditions compared to those women diagnosed with an advanced stage cancer and dying early of the disease. This may introduce a selection bias in the analysis if it is not taken into account in follow-up time, a problem we avoided here.

Women using anti-HT drugs are at a higher risk for cardiovascular morbidity and mortality as compared to non-users. The higher cardiovascular mortality among medication users may appear to modify their observed cancer mortality, creating a bias that favours medication users; in simple terms, if the cardiovascular mortality is high among anti-HT users then their cancer mortality may appear to be low as these women succumb to cardiovascular causes before dying of cancer. We controlled for this possible bias in the competing risk regression model. This analysis confirmed the bias: the risk decrease for all other drug groups except for the ACE-inhibitors became diminished to non-significance. However even in this analysis, the risk decrease among ACE-inhibitor users remained evident, providing further support for the benefits of this drug group.

One previous study has reported a decreased risk of OC death among post-diagnostic ACE-inhibitor users [12]. Previous investigators have also reported better OC survival among beta-blocker users [10,11,12,13]. The follow-up times in these studies have varied between 6 and 15 years with no restrictions for follow-up time or separate analyses for 5-year mortality being applied. Our study does not support a decreased risk of OC death among beta-blocker or ACE-inhibitor users with a five-years follow-up after the OC diagnosis. With a longer follow-up of over ten years, a risk decrease with high-intensity use was observed for several drug groups. When one observes the same trends of risk estimates for multiple anti-HT drug groups with different mechanisms of action, this suggests that the common underlying indication of all of these drugs, i.e., hypertension, may be behind the risk association rather than a direct mechanism of any particular drug group. In our study, furosemide and other diuretics associated with decreased OC survival whereas previous investigators have not evaluated the risk association for these drug groups.

Both ACE-inhibitors and ATR-blockers affect the renin-angiotensin aldosterone system (RAA-system) which controls blood pressure and electrolyte balance in the kidneys. ACE-inhibitors block the whole pathway (activity mediated by both AT_1_- and AT_2_-receptors) by inhibiting the formation of these receptors’ natural agonist, angiotensin II. In contrast, ATR-blockers block only the AT_1_-receptor, just one of the targets of angiotensin II with other receptors such as AT_2_-receptors left unaffected. The stronger risk association observed for ACE-inhibitors might indicate that blockade of the AT_2_-receptor (which ATR-blockers do not antagonize) might have some prognostic value. One can speculate that the RAA-system may have a role in OC progression.

Furosemide use associated with an increased risk of OC death; this drug was analysed separately as it is not primarily used for hypertension control but rather in the management of oedema, for example in cases of heart failure or advanced cancer. It was included, nevertheless, to estimate the possible role of diuretic use in general. We noted that especially a short duration furosemide use associated with an increased risk of OC death. This might be explained by underlying oedema in terminal cancer which is managed with short time furosemide administration. In chronic conditions like heart failure, furosemide is used for longer treatment periods and at higher doses; therefore risk estimates for long-term/high-dose use are less likely to be affected by this kind of confounding by indication, as was observed in our study.

The strengths of our study are the utilization of detailed and reliable national cancer and prescription databases which cover all OC cases during the follow-up period and include all anti-HT drugs purchased by these OC patients during the same time period [20,27]. We were able to take into account simultaneous use of multiple anti-HT drugs which is very common in clinical practice and also the administration of statins and antidiabetic drugs which might also have a prognostic role and thus serve as confounders. We also adjusted for primary OC treatment, age and tumor extent at diagnosis and family history for OC or BCa to control for a selection bias. Instead of evaluating only anti-HT user status, we also assessed cumulative dose, duration and intensity of anti-HT drug use; this represents a major difference and a clear advantage when compared to previous studies. By being able to analyze simultaneously multiple anti-HT drug groups, we could estimate the prognostic role of hypertension management in general versus the use of a particular drug group with a distinctive mechanism of action.

Our follow-up time was also long enough to allow us to estimate separately short-term and long-term mortality, which is essential in OC which often has a poor short-term prognosis.

We did not have information if the anti-HT drugs were actually consumed as we only had information on purchases. Further, we did not have information on blood pressure levels of the participants or indications for medication use, which might have affected the results if hypertension is an independent prognostic factor. We did not have information on the specific indication of anti-HT drug use; for example, ACE-inhibitors, ATR-blockers, beta-blockers and diuretics are first line treatments also in coronary artery disease and heart failure even without hypertension. However, we were able to control for this potential bias by evaluating for cardiovascular mortality among anti-HT drug users. We did not have direct information on possible comorbidities but we could control for comorbidities by taking into account simultaneous use of multiple drugs as a surrogate. In addition, the adjustment for OC treatment also partly adjusts for comorbidities as they may limit the possibilities for curative surgery. The HR for the highest intensity of post-diagnostic ACE-inhibitor use with full follow-up was 0.18 (95% CI: 0.08–0.37) which is a very low value and may be indicative of a selection bias. However, it is unclear what kind of bias would affect only high-intensity users but not low intensity ACE-inhibitor users. We also did not have information on OC progression and possible treatments after OC diagnosis which may cause systematic difference between users and non-users.

## 5. Conclusions

In conclusion, the use of anti-HT drugs does not associate with 5-year OC mortality, with the exception of diuretics. Follow-up time modifies the risk associations; with a longer follow-up, high-intensity use of several drug groups associates with improved survival. When the effect of cardiovascular mortality is taken into account, only ACE-inhibitors are associated with improved OC survival in the long term. If confirmed in further studies, ACE-inhibitors may provide a novel way to exert a positive impact on OC mortality.

## Figures and Tables

**Table 1:** Population characteristics.

**(A)**							
**Characteristics of the Study Population according to Their Pre-Diagnostic Antihypertensive Drug Use.**							
	Non-Users *	**ACE-Inhibitors**	**ATR-Blockers**	**Beta-Blockers**	**Calcium-Channel Blockers**	**Furosemide**	**Other Diuretics**
*n* of women	6841	2217	1144	3072	1889	1139	2800
*n* of biological children, median (IQR)	2 (0–13)	2 (0–12)	2 (0–8)	2 (0–12)	2 (0–10)	2 (0–10)	2 (0–10)
*n* of 1st degree relatives with OC or BCa							
0	6490	2125	1091	2947	1813	1097	2684
1	325	87	50	119	70	40	107
2 or over	26	5	3	6	6	2	9
Median follow up time, years (IQR)	4.4 (0–19)	2.4 (0–18)	2.0 (0–17)	2.1 (0–18)	1.8 (0–18)	0.8 (0–18)	1.9 (0–18)
*n* of OC deaths (% of users)	2485 (36)	879 (40)	431 (38)	1427 (46)	908 (48)	633 (56)	1340 (48)
*n* of all deaths (% of users)	3140 (46)	1134 (49)	559 (36)	1825 (59)	1179 (62)	820 (72)	1737 (62)
Age at diagnosis, median (IQR)	58 (0–113)	70 (18–101)	70 (27–101)	71 (25–101)	73 (30–98)	78 (22–101)	72 (29–101)
Tumor extent at diagnosis, *n* (%)							
Local	2357 (34)	630 (28)	280 (24)	757 (25)	416 (22)	229 (20)	660 (24)
Locally advanced	3050 (45)	944 (43)	526 (46)	1454 (47)	912 (48)	507 (45)	1339 (48)
Advanced	492 (7.2)	274 (12)	156 (14)	372 (12)	250 (13)	181 (16)	332 (12)
Unknown	631 (9.2)	289 (13)	133 (12)	378 (12)	246 (13)	186 (16)	368 (13)
Radical surgery, *n* (% of users)	2548 (37)	637 (29)	305 (27)	781 (25)	462 (24)	237 (21)	728 (26)
Cytostatic therapy, *n* (% of users)	2951 (43)	817 (37)	416 (36)	1147(37)	684 (36)	312 (27)	1019 (36)
Antihormonal therapy, *n* (% of users)	103 (1.5)	29 (1.3)	9 (0.8)	31 (1.0)	23 (1.2)	17 (1.5)	41 (1.5)
Statin use, *n* (% of users)	1144 (17)	1140 (51)	612 (53)	1470 (48)	899 (48)	448 (39)	1242 (44)
Antidiabetic medications; *n* (% of users)	453 (6.6)	656 (30)	303 (26)	714 (23)	464 (25)	322 (28)	715 (26)
**(B)**							
**Characteristics of the Study Population Subdivided by Post-Diagnostic Antihypertensive Drug Use**							
	Non-Users *	**ACE-Inhibitors**	**ATR-Blockers**	**Beta-Blockers**	**Calcium-Channel Blockers**	**Furosemide**	**Other Diuretics**
*n* of women	6810	1525	1005	2757	1395	1380	2292
*n* of biological children, median (IQR)	2 (0–13)	2 (0–12)	2 (0–8)	2 (0–10)	2 (0–10)	2 (0–10)	2 (0–11)
*n* of 1st degree relatives with OC or BCa							
0	6450	1468	962	2648	1337	1321	2203
1	332	54	41	102	53	54	83
2 or over	28	3	2	7	5	5	6
Median follow up time, years (IQR)	4.3 (0–19)	2.9 (0–19)	2.3 (0–17)	2.3 (0–19)	2.2 (0–19)	1.2 (0–19)	2.3 (0–19)
*n* of OC deaths (% of users)	2450 (36)	575 (38)	355 (35)	1254 (45)	617 (44)	829 (560)	1126 (49)
*n* of all deaths (% of users)	3045 (45)	770 (50)	462 (46)	1658 (60)	834 (60)	1068 (77)	1447 (63)
Age at diagnosis, median (IQR)	58 (0–113)	69 (18–101)	69 (29–101)	71 (22–101)	72 (30–98)	77 (22–101)	71 (21–101)
Tumor extent at diagnosis, *n* (%)							
Local	2357 (35)	487 (32)	265 (26)	693 (25)	345 (25)	225 (16)	541 (24)
Locally advanced	2937 (43)	635 (42)	453 (45)	1339 (49)	660 (47)	751 (54)	1170 (51)
Advanced	506 (7.4)	159 (10)	140 (14)	321 (12)	171 (12)	184 (13)	251 (11)
Unknown	691 (10)	193 (13)	108 (11)	309 (11)	167 (12)	170 (12)	250 (11)
Radical surgery, *n* (% of users)	2539 (37)	487 (32)	285 (28)	744 (27)	383 (27)	250 (18)	623 (27)
Cytostatic therapy, *n* (% of users)	2757 (40)	614 (40)	396 (39)	1109 (40)	548 (39)	511 (37)	984 (43)
Antihormonal therapy, *n* (% of users)	90 (1.3)	30 (2.0)	7 (0.7)	37 (1.3)	25 (1.8)	23 (1.7)	33 (1.4)
Statin use, *n* (% of users)	1205 (18)	756 (50)	547 (54)	1321 (48)	642 (46)	453 (33)	920 (40)
Antidiabetic medication; *n* (% of users)	467 (6.8)	464 (30)	275 (27)	662 (24)	343 (25)	321 (23)	562 (25)

IQR = interquartile range, *n* = number, locally advanced: advanced only to regional lymph nodes, advanced: advanced widely to other locations as well as regional lymph nodes. OC = ovarian cancer. BCa = breast cancer. * = women who have not used any anti-HT drugs during the follow-up time.

**Table 2:** Risk of ovarian cancer death by antihypertensive drug use before OC diagnosis.

Drug Group	*n* of Users/OC Deaths	Risk of OC Death, Follow-Up Time		
		0–5 Years	0–10 Years	0–18.9 Years
		HR (95% CI) Multiavariable Adjusted *	HR (95% CI) Multiavariable Adjusted *	HR (95% CI) Multiavariable Adjusted *
ACE inhibitors	2217/879	1.06 (0.98–1.14)	0.92 (0.87–0.98)	0.91 (0.82–1.01)
Intensity of ACE-inhibitor use (DDDs/year)				
Low intensity	740/296	1.08 (0.97–1.21)	1.01 (0.92–1.10)	0.94 (0.80–1.10)
Moderate intensity	738/338	1.05 (0.94–1.17)	0.94 (0.86–1.03)	0.96 (0.83–1.12)
High intensity	739/245	1.03 (0.91–1.16)	0.84 (0.77–0.92)	0.84 (0.71–0.99)
ATR-blockers	1144/431	0.95 (0.87–1.04)	1.00 (0.93–1.08)	0.96 (0.84–1.10)
Beta-blockers	3072/1427	1.02 (0.95–1.09)	1.03 (0.98–1.09)	1.15 (1.05–1.25)
Calcium-channel blockers	1889/908	0.99 (0.91–1.07)	1.07 (1.01–1.14)	1.07 (0.97–1.19)
Furosemide	1139/633	1.25 (1.14–1.38)	1.26 (1.16–1.36)	1.35 (1.20–1.53)
Other diuretics	2800/1340	0.99 (0.92–1.06)	1.07 (1.01–1.14)	1.11 (1.01–1.21)

OC = ovarian cancer, ACE = angiotensin-converting enzyme, ATR = angiotensin-receptor, DDD = defined daily dose, HR = hazard ratio, CI = confidence interval. * = calculated Cox regression model with adjustments of age at diagnosis, year of OC diagnosis, tumor extent, surgery, cytostatic therapy, antihormonal therapy, radiation therapy, number of biological children, age at first labour, number of 1st degree relatives with OC or breast cancer and compared to non-users.

**Table 3:** Risk of OC death according to antihypertensive drug use after the OC diagnosis.

Antihypertensive Drug Group	*n* of Users/OC Deaths	*n* of Person Years	Risk of OC Death, Follow-Up Time		
			0–5 Years	0–10 Years	0–18.9 Years
Drug Group, Intensity of Use (DDDs/year)	*n* of Users/*n* of OC Deaths	*n* of Person Years	HR (95% CI) Multiavariable Adjusted *	HR (95% CI) Multiavariable Adjusted *	HR (95% CI) Multiavariable Adjusted *
ACE inhibitors			1.00 (0.92–1.09)	0.87 (0.81–0.92)	0.81 (0.71–0.93)
Low intensity	743/210	0–15	1.13 (1.02–1.24)	1.03 (0.96–1.11)	0.94 (0.83–1.06)
Moderate intensity	740/197	0–14	0.82 (0.72–0.92)	0.81 (0.74–0.87)	0.67 (0.57–0.79)
High intensity	742/285	0–14	0.81 (0.62–1.06)	0.71 (0.59–0.84)	0.18 (0.08–0.37)
ATR blockers			0.95 (0.87–1.04)	0.90 (0.84–0.98)	0.99 (0.91–1.08)
Low intensity	634/114	0–14	0.96 (0.86–1.08)	0.94 (0.87–1.02)	0.90 (0.78–1.05)
Moderate intensity	634/85	0–13	0.91 (0.80–1.03)	0.86 (0.79–0.94)	0.62 (0.51–0.76)
High intensity	631/227	0–4	0.86 (0.69–1.07)	0.85 (0.72–1.01)	0.42 (0.26–0.68)
Beta-blockers			0.99 (0.92–1.06)	0.98 (0.93–1.03)	0.97 (0.87–1.08)
Low intensity	1385/416	0–14	1.10 (1.02–1.19)	1.09 (1.03–1.15)	1.22 (1.11–1.34)
Moderate intensity	1382/396	0–14	0.84 (0.77–0.92)	0.94 (0.89–1.00)	0.85 (0.76–0.95)
High intensity	1382/710	0–14	0.81 (0.65–1.02)	0.81 (0.68–0.97)	0.64 (0.47–0.88)
Calcium-channel blockers			0.91 (0.84–0.99)	1.04 (0.97–1.11)	1.39 (1.25–1.54)
Low intensity	796/270	0–13	1.11 (0.99–1.24)	1.15 (1.06–1.25)	1.19 (1.04–1.37)
Moderate intensity	794/182	0–8	0.86 (0.77–0.95)	0.96 (0.89–1.04)	0.83 (0.73–0.96)
High intensity	792/283	0–12	0.85 (0.71–1.01)	0.98 (0.86–1.13)	0.67 (0.50–0.90)
Furosemide			1.13 (1.04–1.22)	1.34 (1.25–1.44)	1.06 (0.97–1.16)
Low intensity	928/475	0–18	1.43 (1.31–1.56)	1.66 (1.55–1.78)	2.52 (2.27–2.78)
Moderate intensity	692/299	0–14	1.27 (1.14–1.42)	1.46 (1.34–1.59)	1.78 (1.57–2.03)
High intensity	929/430	0–13	0.98 (0.86–1.13)	1.27 (1.14–1.41)	1.32 (1.10–1.57)
Other diuretics			0.98 (0.91–1.05)	1.07 (1.01–1.13)	1.39 (1.22–1.59)
Low intensity	1221/612	0–18	1.18 (1.08–1.28)	1.25 (1.18–1.33)	1.58 (1.44–1.74)
Moderate intensity	1163/383	0–15	0.89 (0.80–0.98)	1.01 (0.94–1.09)	1.00 (0.88–1.14)
High intensity	1186/515	0–13	1.03 (0.88–1.19)	1.00 (0.89–1.11)	0.95 (0.77–1.17)

OC = ovarian cancer, ACE = angiotensin-converting enzyme, ATR = angiotensin-receptor, DDD = defined daily dose, HR = hazard ratio, CI = confidence interval. * = calculated Cox regression model with adjustments for age at diagnosis, year of OC diagnosis, tumor extent, surgery, cytostatic therapy, antihormonal therapy, radiation therapy, number of biological children, age at first labour, number of 1st degree relatives with OC or breast cancer and compared to non-users.

**Table 4:** Risk of OC death according to antihypertensive use after OC diagnosis. Use of antihypertensive drugs lagged for one, three and five years.

Antihypertensive Drug Group	Risk of OC Death, Follow-Up Time		
	0–5 Years	0–10 Years	0–18.9 Years
Drug Group, Lag-Time (Years)	HR (95% CI) Multiavariable Adjusted *	HR (95% CI) Multiavariable Adjusted *	HR (95% CI) Multiavariable Adjusted *
ACE inhibitors			
1	1.04 (0.96–1.14)	0.89 (0.83–0.94)	0.82 (0.73–0.91)
3	1.02 (0.93–1.12)	0.86 (0.81–0.92)	0.74 (0.65–0.84)
5	1.03 (0.92–1.14)	0.85 (0.79–0.91)	0.74 (0.64–0.86)
ATR blockers			
1	0.98 (0.89–1.07)	0.93 (0.86–1.00)	0.87 (0.76–1.00)
3	1.03 (0.93–1.15)	0.99 (0.91–1.08)	0.98 (0.84–1.15)
5	1.14 (0.98–1.33)	1.09 (0.98–1.21)	0.95 (0.75–1.20)
Beta-blockers			
1	1.04 (0.97–1.11)	1.01 (0.96–1.07)	1.05 (0.96–1.14)
3	1.04 (0.97–1.12)	1.00 (0.94–1.06)	1.06 (0.96–1.17)
5	1.01 (0.93–1.11)	0.97 (0.91–1.03)	1.06 (0.94–1.19)
Calcium-channel blockers			
1	0.96 (0.89–1.04)	1.08 (1.01–1.15)	1.06 (0.95–1.18)
3	0.95 (0.87–1.04)	1.06 (0.98–1.14)	1.09 (0.97–1.23)
5	0.99 (0.89–1.11)	1.04 (0.96–1.13)	1.11 (0.96–1.28)
Furosemide			
1	1.16 (1.06–1.26)	1.35 (1.25–1.46)	1.42 (1.27–1.59)
3	1.04 (0.93–1.18)	1.16 (0.98–1.14)	1.19 (1.03–1.39)
5	1.00 (0.82–1.22)	0.93 (0.82–1.05)	1.04 (0.84–1.29)
Other diuretics			
1	0.99 (0.92–1.06)	1.06 (1.00–1.13)	1.07 (0.98–1.17)
3	0.93 (0.86–1.01)	1.01 (0.95–1.07)	0.98 (0.88–1.09)
5	0.95 (0.86–1.05)	1.01 (0.94–1.09)	1.08 (0.95–1.23)

OC = ovarian cancer, ACE = angiotensin-converting enzyme, ATR = angiotensin-receptor, DDD = defined daily dose, HR = hazard ratio, CI = confidence interval, * = calculated Cox regression model with adjustments for age at diagnosis, year of OC diagnosis, tumor extent, surgery, cytostatic therapy, antihormonal therapy, radiation therapy, number of biological children, age at first labour, number of 1st degree relatives with OC or breast cancer and compared to non-users.

## Data Availability

No new data were created or analyzed in this study. Data sharing is not applicable to this article.

## Supplementary Materials

The following are available online at https://www.mdpi.com/article/10.3390/cancers13092087/s1, Table S1: ATC-codes for antihypertensive drugs, Table S2: Risk of OC death by antihypertensive drug use. A competing risk analysis.
