# Supplementary material for: Antihypertensive Drug Use and the Risk of Ovarian Cancer Death among Finnish Ovarian Cancer Patients—A Nationwide Cohort Study
Source: Cancers (Basel). 2021 Apr 26;13(9):2087. doi: 10.3390/cancers13092087 (PMC8123393; doi:10.3390/cancers13092087)
Supplement: Supplementary file 1 [file cancers-13-02087-s001.zip › cancers-1172808-supplementary.pdf]

cancers

MDPI

Article

Supplementary Materials: Antihypertensive Drug Use and the Risk of Ovarian Cancer Death among Finnish Ovarian Cancer Patients—A Nationwide Cohort Study

Eerik E.E. Santala, Miia Artama, Eero Pukkala, Kala Visvanathan, Synnöve Staff, Teemu J. Murtola

Table S1. ATC-codes for antihypertensive drugs

Drug	ATC-Code
Enalapril	C09AA02, C09BA02, C09BB02
Imidapril	C09AA16
Captopril	C09AA01, C09AB01
Cinapril	C09AA06, C09BA06
Lisinopril	C09AA03, C09BA03
Perindopril	C09AA04, C09BA04, C09BB04
Ramipril	C09AA05, C09BA05, C09BB05
Trandolapril	C09AA10, C09BB10
Other ACE-inhibitors	C09AA08, C09BA08, C09AA07, C09BA07, C09AA09, C09BA09, C09AA11, C09AA12, C09BA12, C09BB12, C09AA13, C09BA13, C09AA14, C09AA15, C09BA15
Eprosartan	C09CA02, C09DA02
Candesartan	C09CA06, C09DA06
Losartan	C09CA01, C09DA01
Olmesartan	C09CA08, C09DA08, C09DB02
Telmisartan	C09CA07, C09DA07
Valsartan	C09CA03, C09DA03, C09DB01, C09DX01
Irbesartan	C09CA04, C09DA04
Other sartans	C09CA05
Clonidine	C02AC01, C02LC01, C02LC51
Moxonidine	C02AC05, C02LC05
Rauwolfia-alkaloids	C02AA01, C02AA02, C02AA03, C02AA04, C02AA05, C02AA06, C02AA07, C02AA52, C02AA53, C02AA57
Other antagonists of imidazoline receptor	C02AC02, C02AC04, C02AC06
Antiadrenergics	C02BA01, C02BB01
Prazosine	C02CA01, C02LE01
Other alfa-blocking antihypertensives	C02CA02, C02CA03, C02CA04, C02CA06
Guanidines	C02CC01, C02CC02, C02CC03, C02CC04, C02CC05, C02CC06, C02CC07, C02LF01
Smooth muscle relaxants	C02DA01, C02DB01, C02DB02, C02DB03, C02DB04, C02DD01, C02DG01
Other antihypertensives	C02KA01, C02KB01, C02KC01, C02KD01
Acebutolol	C07AB04, C07BB04
Atenolol	C07AB03, C07BB03, C07CB03, C07CB53, C07DB01, C07FB03
Betaxolol	C07AB05
Bisoprolol	C07AB07, C07BB07

Carvedilol	C07AG02
Labetalol	C07AG01, C07BG01, C07CG01
Metoprolol	C07AB02, C07AB52, C07BB02, C07BB52, C07CB02, C07FB02
Pindolol	C07AA03, C07CA03
Propranolol	C07AA05, C07BA05, C07FA05
Seliprolol	C07AB08
Timolol	C07AA06, C07BA06, C07DA06
Nebivolol	C07AB12
Other beta-blockers	C07AA01, C07AA12, C07AA14, C07AA15, C07AA16, C07AA17, C07AA19, C07AA23, C07AA27, C07AB01, C07AB06, C07AB09, C07AB10. C07AB11, C07AB13, C07BA12, C07BA68, C07BB06, C07CA17, C07CA23
Amiloride	C03DB01, C03EA01
Furosemide	C03CA01, C03CB01, C03EB01
Hydrochlorothiazide	C03AA03, C03AB03, C03AX01, C02LB01, C02LC01, C02LC05, C02LC51, C02LE01, C07BA02, C07BA05, C07BA06, C07BA07, C07BA12, C07BA68, C07BB02 C07BB03, C07BB04, C07BB06, C07BB07, C07BB52, C07BG01, C07DA06, C07DB01 C08GA01, C09BA01, C09BA02, C09BA03, C09BA05, C09BA06, C09BA07, C09BA08, C09BA09, C09BA12, C09BA13, C09BA15, C09DA01, C09DA02, C09DA03 C09DA04, C09DA06, C09DA07 C09DA08, C03EA01
Indapamide	C03BA11, C09BA04
Spironolactone	C03DA01
Triamteren	C03DB02, C03EA02, C03EA03, C03EA04, C03EA05, C03EA06, C03EA07, C03EA12, C03EA13, C03EA14, C03EB01, C03EB02
Trichlormethiazide	C03AA06, C03AB06, C03EA02
Other diuretics	C03AA01, C03AA02, C03AA04, C03AA05, C03AA07, C03AA08, C03AA09,C03AA13, C03AB01, C03AB02, C03AB04,C03AB05, C03AB07, C03AB08, C03AH01, C03AH02, C03BA02, C03BA03, C03BA04, C03BA07, C03BA09, C03BA10, C03BA12,C03BA13, C03BA82, C03BB02, C03BB03, C03BB04, C03BB07, C03BC01, C03BD01, C03BX03, C03CA03, C03CA04, C03CC01,C03CC02, C03CD01, C03CX01, C03DA02, C03DA03, C03DA04, C03EA03, C03EA04, C03EA05, C03EA06, C03EA07, C03EA13, C03EA14, C03XA01, C03XA02
Amlodipine	C08CA01, C09DB01, C09DB02, C09DX01, C09BB04
Diltiazem	C08DB01
Felodipine	C08CA02, C09BB05, C07FB02
Isradipine	C08CA03
Lercanidipine	C08CA13, C09BB02
Nifedipine	C08CA05, C08CA55, C08GA01, C07FB03
Nilvadipine	C08CA10
Nimodipine	C08CA06
Nisoldipine	C08CA07

Verapamil	C08DA01, C08DA51, C09BB10
Other calcium-channel blockers	C08CA04, C08CA08, C08CA09, C08CA11, C08CA12, C08CA14, C08CA15, C08CX01, C08DA02, C08EA01, C08EA02, C08EX01, C08EX02, C09BB12
Aliskiren	C09XA02

Table S2. Risk of OC death by antihypertensive drug use. A competing risk analysis.

Drug Group	Risk of OC Death, HR (95% CI)
ACE inhibitors	0.73 (0.58–0.91)
ATR-blockers	0.62 (0.34–1.12)
Beta-blockers	0.86 (0.72–1.02)
Calcium-channel blockers	0.93 (0.71–1.21)
Furosemide	1.03 (0.85–1.25)
Other diuretics	1.03 (0.81–1.32)
